# Human Chorionic Gonadotropin as a Marker of Parathyroid Carcinoma: Findings From a Scoping Review

**DOI:** 10.7759/cureus.86141

**Published:** 2025-06-16

**Authors:** Maxim J Barnett, Sarah Eidbo, Carlo Casipit, Justin Lam, Catherine Anastasopoulou

**Affiliations:** 1 Internal Medicine, Jefferson Einstein Philadelphia Hospital, Philadelphia, USA; 2 Endocrinology, Diabetes and Metabolism, Jefferson Einstein Philadelphia Hospital, Philadelphia, USA

**Keywords:** beta-hcg, biomarker testing, hcg, human chorionic gonadotropin (hcg), parathyroid cancer

## Abstract

Parathyroid carcinoma is an extremely uncommon cause of primary hyperparathyroidism; however, it is associated with excess morbidity and mortality. With the similarity in presentation from benign etiologies (such as an adenoma or hyperplasia), there is often a delay in diagnosis and initiation of treatment. Currently, there are no approved biomarkers to assist in the diagnosis of carcinoma. Over the last century, limited studies have investigated the role of human chorionic gonadotropin (hCG), particularly in its ability to differentiate cancerous from benign parathyroid disease. We performed a scoping review to analyze the existing literature regarding hCG (serum, urine, and histochemical staining) and its role in identifying malignant parathyroid disease. Across three databases and an endocrine abstract repertoire, we identified seven relevant articles meeting the prespecified inclusion criteria. We highlighted the normal physiology of hCG, alongside the pathophysiological expression and the rationale for its use as a biomarker for parathyroid cancer. Each article was analyzed, with emphasis on the methodologies, outcomes, and limitations. Although promising, the role of hCG as a biomarker has not been established due to heterogeneous methodologies and limited studies, which prohibit a firm conclusion. Further research (including larger prospective studies) is required to validate the role of hCG as a biomarker for parathyroid carcinoma.

## Introduction and background

Parathyroid cancer is an infrequent yet perilous diagnosis, accounting for no more than 1% of all cases of primary hyperparathyroidism [1]. The first case of parathyroid carcinoma was diagnosed in 1904 by de Quervain, with no more than a few thousand cases described since then [2]. Diagnosis is often delayed due to the low levels of suspicion and is often secondary to a lack of specific features preoperatively [3]. Suspicious findings include markedly elevated calcium and parathyroid hormone (PTH) levels, end-organ damage, or unusually large parathyroid tumors, with more than three-quarters of patients having a palpable neck mass on physical examination (on average above 3 cm in size) [4,5].

Clinicians should be suspicious for parathyroid cancer when PTH is more than three times the upper limit of normal (occasionally being as high as 10-15 times the upper limit of normal with levels above 1,000 pg/mL or 14-15 mg/dL) [5-7]. In contrast to primary hyperparathyroidism (female to male ratio of 3-4:1), there is an equal sex preponderance with parathyroid carcinoma, with an average age of diagnosis around 10 years earlier than with benign primary hyperparathyroidism (mid-40s compared to mid-50s) [5]. Unfortunately, there are no pathognomonic findings, and the diagnosis is often made retrospectively once hypercalcemia recurs due to local spread of tumor or distant metastases [8]. In rare cases, parathyroid cancer may be non-functional and is often misdiagnosed [6].

*CDC73* (*HRPT2*) mutations are frequently identified and believed to play a pathologic role in the molecular mechanism. This gene is found on chromosome 1 and encodes for parafibromin, which is involved in hyperparathyroidism-jaw tumor syndrome, a syndrome associated with a 15% risk for parathyroid carcinoma [9,10]. Up to 70% of sporadic parathyroid carcinomas demonstrate *CDC73* mutations (compared to 2% of parathyroid adenomas) [11]. Less commonly, multiple endocrine neoplasia has also been described with an association with parathyroid carcinoma [12]. The chance for a cure is associated with complete resection at the time of the initial surgery, with an en-bloc resection still recommended and considered the gold standard [13]. The current five-year overall survival ranges from 60% to 93%, with a recurrence rate of 50% [14].

Previously, human chorionic gonadotropin (hCG; both serum and urine) has been investigated as a tumor marker in parathyroid carcinoma, differentiating from benign primary hyperparathyroidism. The elevated hCG isotype appears to be the hyperglycosylated form, which is specifically found with malignancy in trophoblastic and non-trophoblastic diseases. We performed a scoping review to analyze and investigate the current literature examining the role of hCG as a tumoral marker for parathyroid carcinoma.

## Review

Methodology

Protocol and Framework

The Joanna Briggs Institute methodology for scoping reviews was utilized for this review [15]. Furthermore, to ensure transparency and comprehensive reporting, the Preferred Reporting Items for Systematic Reviews and Meta-Analyses Extension for Scoping Reviews (PRISMA-ScR) checklist was used [16]. Our primary objective was to explore the literature regarding the role of hCG as a biomarker for parathyroid cancer; specifically, our review aimed to answer the question: “What evidence exists regarding the use of hCG as a diagnostic, prognostic, or monitoring biomarker in patients with parathyroid carcinoma?”

Eligibility Criteria

This scoping review incorporated the PCC (Population-Concept-Context) framework to define the inclusion and exclusion criteria (Table 1) [15]. The population chosen for this study was patients with a confirmed or suspected diagnosis of parathyroid carcinoma, with no age or gender restriction. Our concept was studies that reported either hCG or its subunits as a biomarker (either as immunohistochemical staining, urinary, or serum measurements). The context included all healthcare settings, without geographic, language, or time restrictions, and all articles were given full consideration.

**Table 1:** Population-Concept-Context framework. hCG: human chorionic gonadotropin

	Inclusion	Exclusion
Population	Patients with confirmed (or suspected) parathyroid carcinoma. All genders and ages	Studies not involving parathyroid carcinoma. Studies not involving humans
Concept	Studies reporting on hCG (or its subunits, such as hyperglycosylated hCG, alpha-hCG, or beta-hCG) as a biomarker. Immunohistochemical staining, urinary measurements, or serum measurements	Reports failing to investigate hCG (or its subunits)
Context	All healthcare settings and all article types. No geographic, language, or time restriction	-

Results

Search Strategy

A literature search was performed across PubMed/MEDLINE, Cumulative Index to Nursing and Allied Health Literature (CINAHL; to include conference proceedings and grey literature which would otherwise be missed), Cochrane Register of Controlled Trials (CENTRAL; for small/unpublished trials which may be examining hCG as a biomarker), and Endocrine Abstracts Conference Proceedings (to further identify grey literature which could be missed). Our search was conducted up to May 20, 2025, with no limits placed on the date of publication. References of articles meeting the inclusion criteria were also screened (manually) to identify additional relevant studies.

Study Selection

**Figure 1:**
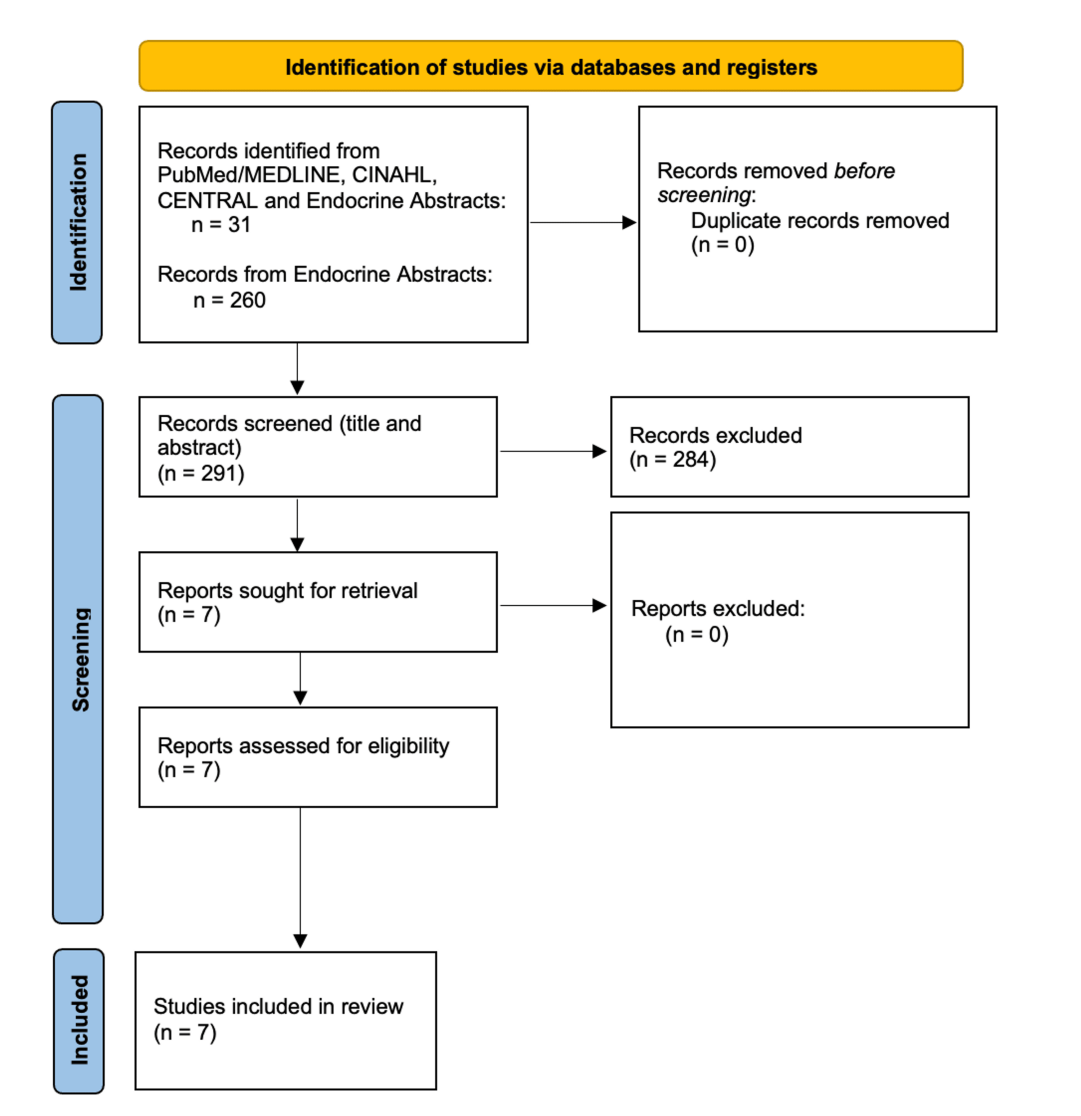
Preferred Reporting Items for Systematic Reviews and Meta-Analyses (PRISMA) search strategy.

A comprehensive search was performed across the three databases, which identified a total of 31 studies by creating a search string “hCG and parathyroid.” All retrieved records were imported into Microsoft Excel to compare. No duplicates were identified (n = 0). All authors independently screened the remaining titles and abstracts. In total, 25 were removed as they failed to meet the inclusion criteria. Full-text screening of the remaining six studies resulted in all meeting the inclusion criteria [17-23]. Upon a review of Endocrine Abstracts, an initial search demonstrated 260 studies; however, after screening the titles and abstracts, only one study fulfilled the inclusion criteria and was incorporated into our scoping review. Figure 1 demonstrates the selection process, with a total of seven studies included in this review.

Data Extraction

Standardized data extraction included a table with publication details (author(s), year published, country), study design, sample size, characteristics of the participants, hCG type reported, assay method, and key findings from the study (Table 2) [17-23]. In addition to the tabular format of data presented, findings are discussed narratively with descriptive numerical summaries of included studies. A thematic analysis was performed to identify and organize patterns related to the utility of hCG.

**Table 2:** Characteristics of the included studies. hCG: human chorionic gonadotropin; MEN: multiple endocrine neoplasia; PTH: parathyroid hormone

Authors	Year	Country	Study design	Number of participants	hCG isoform	Sample type	Main findings
Stock et al. [17]	1983	USA	Observational	Carcinoma: n = 3. Benign: n = 42. MEN: n = 30	Alpha- and beta-hCG	Serum, gland effluent, and tissue extract	Serum alpha- and beta-hCG were elevated in two-thirds of carcinoma patients (normalizing postoperatively). In one of the patients with malignancy, beta-hCG rose with disease progression
Chng et al. [18]	1990	Malaysia	Case report	Carcinoma: n = 1	Beta-hCG	Serum	Elevated beta-hCG in a male patient, which demonstrated a significant positive correlation with PTH, both of which normalized postoperatively
Carlifante et al. [19]	1999	Italy	Observational	Carcinoma: n = 3. Adenoma: n = 31. MEN: n = 16. Other: n = 36	Alpha- and beta-hCG	Tissue	Expression of alpha-hCG was present in 54%, with beta-hCG expressed in 39%. hCG expression was present throughout all forms of hyperparathyroidism. All three patients with parathyroid carcinoma expressed alpha-hCG expression (100%); however, only one-third demonstrated beta-hCG expression (33%)
Rubin et al. [20]	2008	USA	Observational	Carcinoma: n = 8. Benign: n = 18	Hyperglycosylated hCG and total hCG	Serum and urine	Elevated total urinary hCG in five of eight parathyroid carcinoma patients (none with benign hyperparathyroidism). Elevated (or rising) total urinary hCG correlated with survival and complications. All five of the eight patients with elevated total urinary hCG demonstrated elevated urinary hyperglycosylated hCG. In all eight parathyroid carcinoma patients, serum malignant hyperglycosylated hCG exceeded the levels of benign hyperparathyroidism
Gupta et al. [21]	2013	Nepal	Retrospective case-control	Carcinoma: n = 10. Benign: n = 10	Hyperglycosylated hCG and total hCG	Urine	Urinary total hCG was elevated in six of 10 parathyroid carcinoma patients (no benign hyperparathyroidism cases). Malignant (hyperglycosylated) hCG was elevated in four of 10 parathyroid carcinoma patients (no benign hyperparathyroidism cases)
Valdes-Socin et al. [22]	2018	Belgium	Observational	Carcinoma: n = 8. Primary hyperparathyroidism: n = 20. Secondary hyperparathyroidism: n = 25	Beta-hCG	Serum	Mean beta-hCG across eight carcinoma patients was significantly greater than benign hyperparathyroidism cases. A significant positive correlation between hCG and PTH was identified (r = 0.786, p < 0.05)
Yin et al. [23]	2023	China	Case report	Parathyroid carcinoma = 2	Beta-hCG	Serum	Beta-hCG was only measured in one patient (functional parathyroid carcinoma), whereby it was found to be slightly increased, compared to a massive elevation of calcium and PTH

The earliest investigation of the role of hCG for parathyroid disease was reported by Stock et al. [18] in 1982, who studied three patients with parathyroid carcinoma, 42 patients with benign primary hyperparathyroidism, and 30 with multiple endocrine neoplasia, of whom 18 had active primary hyperparathyroidism. Serum alpha- and beta-hCG were elevated in two out of three patients with parathyroid carcinoma, which rapidly decreased following surgical intervention. On follow-up, the beta-hCG began to rise in one of these patients, correlating with disease progression. The authors further analyzed an extract from the parathyroid carcinoma of one patient, noting significant elevations in both alpha- and beta-hCG. While the third patient with parathyroid carcinoma did not demonstrate elevated serum hCG levels, the patient demonstrated drastically lower levels of PTH, raising the possibility of a non-secretory lesion. To assess the specificity of hCG for malignant disease, the authors investigated the benign cases of primary hyperparathyroidism (n = 42), with 37 patients having normal peripheral and gland effluent levels of alpha- and beta-hCG. Of the five patients, four had subunits found to originate outside of the parathyroid glands, and one had sole minimal elevation of alpha-hCG (but not beta) in the parathyroid venous effluent, but not in the serum. Of the 18 patients with active primary hyperparathyroidism and multiple endocrine neoplasia, none demonstrated abnormal peripheral concentrations of hCG subunits. The authors further compared the extract from one patient with parathyroid carcinoma to seven parathyroid glands with benign primary hyperparathyroidism, noting detectable subunits in two patients. Of these, one had elevations in alpha-hCG (but not beta-hCG), and one had measurable beta-hCG (but not alpha-hCG). The latter patient did have a measurable beta-hCG; however, the patient was receiving dialysis, which can lead to accumulation, and was hypothesized to contaminate the parathyroid extract. Stock et al. [18] noted that the subunit concentration of these extracts from the two patients with benign primary hyperparathyroidism was less than 1% of that found in the parathyroid carcinoma extract.

In 1990, Chng et al. [18] presented a young male with primary hyperparathyroidism, found to have parathyroid carcinoma on histopathology. Furthermore, he was found to have elevated serum beta-hCG, which appeared to be steroid-suppressible (alongside PTH), both of which remained suppressed postoperatively. The authors hypothesized that the paradoxical response to steroid suppression with hyperparathyroidism may be a result of the underlying neoplastic process, for which the feedback inhibitory pathway is lost.

Subsequently, in 1999, Carlifante et al. [19] investigated the expression of hCG subunits in parathyroid specimens (encompassing multiple causes of hyperparathyroidism: multiple endocrine neoplasia type 1 and 2, adenoma, uremic secondary hyperparathyroidism, and carcinoma) across 86 patients. Expression of alpha- and beta-hCG was noted in 54% and 39%, respectively, across all patients. Regarding parathyroid carcinoma, alpha-hCG expression was noted in 100% (n = 3) of patients; however, beta-hCG expression was noted in 33% (n = 1). The authors hypothesized that expression of hCG in parathyroid disease can occur in benign conditions; however, benign states exert an efficient control mechanism in the release of such hormones, which is likely defective in carcinomatous states.

The next investigation occurred in 2008 by Rubin et al. [20], who assessed the urine and serum of eight patients with parathyroid carcinoma compared to 18 with benign primary hyperparathyroidism. The authors demonstrated normal total urinary hCG in all 18 patients from the benign cohort. However, abnormal urinary hCG was noted in five patients with parathyroid carcinoma. There appeared to be a significant correlation with complications and mortality in the carcinoma group (three patients with normal urinary hCG were complication free for at least two years; three patients with persistent elevations had hip fractures, with two of these patients dying within six months; and of the two patients with a progressive rise, one had a hip fracture and both died within 10 months). Of the five patients with elevated total urinary hCG in the carcinoma cohort, all demonstrated elevated urinary malignant (hyperglycosylated) hCG (which was undetectable in the remaining three patients who were complication-free, as well as the benign cohort). The authors noted that serum malignant (hyperglycosylated) hCG in the carcinoma cohort exceeded the maximal levels of the benign cohort. From this study, the sensitivity of urinary hCG to discern benign disease from parathyroid carcinoma was posed as 40%, with a specificity of 100%.

Five years later, Gupta et al. [21] compared 10 patients with parathyroid carcinoma to 10 with benign primary hyperparathyroidism, comparing both total and hyperglycosylated urinary hCG. The authors identified six patients in the carcinoma cohort with elevated total urinary hCG (and none in the benign cohort). Furthermore, four patients within the carcinoma cohort demonstrated elevated urinary malignant (hyperglycosylated) hCG (with none from the benign cohort).

In 2018, at the 20th European Endocrine Conference by Valdes-Socin et al. [22], eight patients with parathyroid cancer were compared to 20 with primary hyperparathyroidism and a further 25 with secondary hyperparathyroidism (from chronic renal failure). The authors noted a significant correlation between serum beta-hCG and PTH in the carcinoma cohort (r = 0.786, p < 0.05) and documented a significantly higher median serum beta-hCG in the carcinoma cohort compared to both the primary and secondary hyperparathyroidism cohorts. Valdes-Socin et al. [22] further documented a sensitivity of 75% and specificity of 94% to detect parathyroid cancer using beta-hCG. The authors noted that within the carcinoma cohort, the lowest beta-hCG level was identified in a patient who had biochemical normalization and tumor shrinkage following immunotherapy, for which they hypothesized the role of hCG as a prognostic (in addition to diagnostic) biomarker.

The final report by Yin et al. [23] in 2023 involved an elderly female with a functional parathyroid carcinoma. The authors noted that despite a significant elevation in serum PTH and calcium, there was only a slight increase in the patient’s beta-hCG. It should be noted, however, that there was no further trending postoperatively, nor were other isoforms (or methods of collection, such as urine) performed. While the authors presented a second patient with a non-functional parathyroid carcinoma, hCG was not measured in that patient.

Discussion

In 1927, Ascheim and Zondek first identified hCG, noting that the urine of pregnant women could induce ovulation in immature mice, leading to the development of the first bioassay for pregnancy testing, known as the Ascheim-Zondek test [24]. Placental syncytiotrophoblast is the site of production for hCG and can be detected within days of implantation [25]. In early pregnancy, hCG maintains the corpus luteum, alongside angiogenesis, quiescence of the myometrium, and immunomodulation [26]. Similar to luteinizing hormone, follicle-stimulating hormone, and thyroid-stimulating hormone, hCG is a glycoprotein, composed of a common alpha and beta subunit (the latter conferring biological specificity) [27]. Existing forms of hCG include intact, hyperglycosylated, and beta-subunit. In addition to placental syncytiotrophoblasts, both trophoblastic and non-trophoblastic tumors (such as lymphoma, carcinoid tumor, transitional cell carcinoma, lung, breast, and oral cancers) have been documented to secrete hCG [28].

We investigated the role of hCG as a biomarker in parathyroid carcinoma by performing a scoping review, encompassing seven studies. Of the seven studies, two were case reports, one was presented as an abstract, and the remaining four were observational studies. Our scoping review analyzed data between the years 1982 and 2023, with representation from Belgium (n = 1), China (n = 1), Italy (n = 1), Malaysia (n = 1), Nepal (n = 1), and the United States (n = 2). Sample sizes analyzed ranged from a single patient (case report) to 86 histological samples. When the data of all studies were combined, 34 cases of parathyroid carcinoma were compared to 228 controls (with varying causes of hyperparathyroidism).

Across the seven studies, several key themes were identified. Both serum and urinary hCG (particularly the hyperglycosylated form) appeared to be elevated in parathyroid carcinoma compared to benign disease. While sensitivity was inconsistent, notable specificity was evident in distinguishing malignancy. Similarly, it appears that elevated (or rising) hCG levels may correlate with disease activity and predict worse outcomes, including fracture, recurrence, and mortality, with hCG trends potentially aligning with the response to treatment. Immunohistochemical analysis provided biological plausibility into the mechanisms, by suggesting that the hCG (subunit) production occurs within the parathyroid tissue of both benign and malignant disease, albeit with significant differences in concentration and cellular expression.

The implementation of hCG as a diagnostic biomarker for parathyroid carcinoma was supported by its investigation in all seven studies, albeit to varying degrees. Of these, six studies demonstrated elevated levels in carcinoma compared to benign causes, with one solely analyzing tissue extracts, noting positive expressions in both benign and malignant disease. Total (urinary and serum), hyperglycosylated (urinary and serum), and beta subunit (serum) all consistently noted elevations in parathyroid carcinoma, in addition to expression in tissue extract, suggesting its involvement in malignant transformation rather than an incidental marker. Elevated hyperglycosylated hCG was exclusively identified in patients with parathyroid carcinoma in the studies by Rubin et al. [20] and Gupta et al. [21]. While not all patients with parathyroid carcinoma demonstrate elevated hCG levels, its presence is strongly suggestive of malignancy with strong specificity.

Of the seven studies, three investigated the prognostic (or monitoring) applications of hCG in parathyroid carcinoma. The studies by Rubin et al. [20] and Valdes-Socin et al. [22] suggested that repetitive monitoring of hCG can serve as a prognostication tool, with rising levels correlating with clinical deterioration and adverse events, and normalization predicting favorable survival. As a result, serial measurements may serve as a longitudinal marker for disease activity and efficacy of treatment.

The expression of hCG subunits within the parathyroid gland raises an important question regarding its role in the pathophysiology of underlying disease states. The role of tissue expression was noted in two studies. While Carlifante et al. [19] noted expression among both benign and malignant parathyroid disease, Stock et al. [17] noted that expression was more common in malignant disease (particularly at higher levels). Stock et al. [17] further noted that serum subunit elevation was more common with carcinomatous disease, a finding that suggests a breakdown in the regulatory homeostasis of the cell’s ability to secrete glycoprotein, which is preserved in benign disease.

Unlike established (or emerging) markers of malignant parathyroid disease, such as *CDC73* mutations and loss of parafibromin expression, hCG represents a non-invasive (rapidly available) and practical biomarker, particularly when there is diagnostic uncertainty or biopsy and surgical resection are delayed. Including hCG in a multi-parametric assessment (similar to a thyroid or adrenal nodule workup) alongside radiological, biochemical, and genetic markers can enhance diagnostic accuracy and provide a more comprehensive risk stratification. While tissue staining supports biological plausibility, it lacks real-time diagnostic use and is best correlated with concurrent serum (or urine) measurements. With the variability in sensitivity, however, its use as a screening tool is limited, underscoring the need for interpretation in the broader clinical context.

Strengths and limitations

To our knowledge, this scoping review is the first to systematically explore the role of hCG (and its various isoforms) in the diagnosis of parathyroid carcinoma. Major strengths include the various hCG isoforms (and sources), exploring the expanded role as a potential biomarker. Additionally, our comprehensive search strategy across multiple databases (including grey literature) with no time or language restriction minimized the risk for publication bias. Furthermore, we demonstrated biological plausibility, enhancing the rationale for its role as a biomarker. In addition to a summarization of data, key themes identified by the various articles were discussed, analyzing its practical implications as a diagnostic tool.

Limitations are present, however, including the limited number of studies (n = 7), which is constrained by the rarity of parathyroid carcinoma, leading to difficulty arriving at robust conclusions. Methodologically, study populations and designs were heterogeneous, single-center, and retrospective, introducing substantial selection and measurement bias. Moreover, there was significant variability in assay methods, with a lack of standardization. Similarly, assays have not been validated for parathyroid tissue, with reported assays used from oncology and obstetrics. Differences in immunohistochemistry fixation, interpretation, and antibody specificity complicated the interpretation. While specificity was high for carcinoma, sensitivity was suboptimal, with hCG undetectable in a subset of confirmed cases. Moreover, differences in subunit measurements, sources of hCG, and frequency of measurements limit comparability among studies. Another consideration is that renal function was not reported in all studies, which can alter both urinary (and serum) hCG measurements.

Despite these limitations, however, the results of this scoping review show potential for the role of hCG as a diagnostic and prognostic marker for parathyroid carcinoma. Further validation will require prospective standardized hCG isoform assays to estimate sensitivity, specificity, and predictive values (both positive and negative). Follow-up by longitudinal studies is required to study the trend of hCG before and after surgery on parathyroid carcinoma and measure its response to therapy. Tissue analyses are required to further explore if hCG plays a role in tumorigenesis or metastases. Ultimately, with this data, international consensus will later consider the incorporation of hCG into the diagnostic and follow-up guidelines for parathyroid carcinoma.

## Conclusions

This scoping review highlights an emerging role for hCG, particularly the hyperglycosylated isoform, as a potential biomarker in parathyroid carcinoma. While the data is limited and methodologically heterogeneous, trends suggest diagnostic and potential prognostic value. Elevated hCG levels appear specific for malignancy, albeit not universally present, and may potentially aid in risk stratification in conjunction with additional markers. Given the rarity of parathyroid carcinoma, further studies are required to validate the findings of this scoping review.
